# Interactions and Oscillatory Dynamics of Chemically Powered Soft Swimmers

**DOI:** 10.1021/acs.jpcb.4c07069

**Published:** 2024-12-23

**Authors:** Suzanne Ahmed, Juan Perez-Mercader

**Affiliations:** † Department of Nanoscience, Joint School of Nanoscience and Nanoengineering, University of North Carolina at Greensboro, 2907 E Gate City Blvd, Greensboro, North Carolina 27401, United States; ‡ Department of Earth and Planetary Sciences and Origins of Life Initiative, Harvard University, 20 Oxford Street, Cambridge, Massachusetts 02138, United States; § Santa Fe Institute, Santa Fe, New Mexico 87501, United States

## Abstract

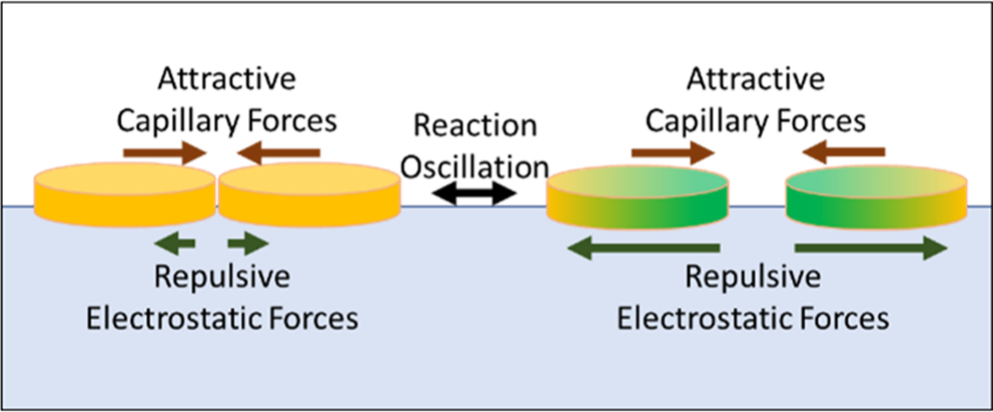

We report the interactions and dynamics of chemically powered soft swimmers that undergo autonomous oscillatory motion. The interaction of autonomous entities is the basis for the development of collective behaviors among biological organisms. Collective behaviors enable organisms to efficiently attain food and coordinate against threats. The basis of these behaviors is the interaction between nearest neighbors. Mimicking these interactions in artificial systems would enable their organization for the performance of complex tasks. Oscillatory phenomena are also ubiquitous in nature. Hence artificial oscillatory systems can serve as the most direct mimics and models of many biological systems. In this work, we report the interactions and dynamics of oscillatory swimmers propelled by the nonlinear oscillatory Belousov–Zhabotinsky (BZ) reaction. Individually, these swimmers displace by undergoing nonfully reciprocal oscillatory motion in conjunction with the BZ reaction. We find that, in addition to their individual oscillatory motion, multiple BZ swimmers exhibit successive oscillatory changes in their inter swimmer distance. This oscillatory attraction and repulsion between adjacent swimmers occurs in conjunction with the BZ waves and oxidation state of the catalyst. The effect of swimmer size and number on these dynamic interactions is interrogated. The level of chemical synchronization between multiple swimmers is determined. This work is a starting point for the design of collective behaviors utilizing autonomous chemically propelled soft swimmers.

## 1 Introduction

The ability of motile biological entities to interact and adapt their motion is essential to their ability to carry out complex functions, such as the escape from threats or the finding of food.^1^ Communication induced collective behaviors is a universal phenomenon observed in the organization of macroscopic and microscopic organisms, such as in the schooling of fish or the swarming of bacteria.^2−4^ These behaviors are mediated at their most basic level via nearest neighbor interactions. The ability to mimic these biological interactions in artificial systems would provide a model to further the understanding of biological systems.^5,6^ It would also allow the harnessing of these systems for use in various applications including crack detection and repair, drug delivery and environmental remediation that will necessarily require multiple structures.^7−14^

Synthetic swimmers propelled using a variety of methods including chemical, electrical, magnetic, acoustic and electromagnetic methods have been developed.^15−24^ The ability of some of these particles to carry out collective behaviors has been demonstrated and the nature of their interactions have been studied.^6,25−36^ Interactions have been studied for both autonomous and nonautonomous swimmers. Autonomous swimmers often rely on the local catalytic decomposition of a fuel allowing each swimmer to act independently of the others, while nonautonomous swimmers tend to move because of an overpowering external field inducing largely identical motion from one swimmer to the next. Autonomous swimmers, as such, more closely mimic the behavior of biological entities. Hence it is the establishment of interactions between these autonomous entities that can give rise to biomimetic collective behaviors.^5^

Autonomous swimmers have been shown to undergo collective behaviors,^6,25−36^ yet the majority of these particles have either been metallic or composed of inorganic materials and have utilized hydrogen peroxide fuels. Soft swimmers more closely model biological systems and are more flexible, maneuverable and potentially biocompatible.^37−39^ Chemically propelled, autonomous soft swimmers that utilize organic fuels are the closest models for biological entities.

Additionally, as oscillatory mechanical behavior is also ubiquitous in biological systems such as in the beating of a heart or the beating of a flagellum to enable the motion of a microorganism, the ability to transduce energy into oscillatory mechanical behavior would provide a more robust biomimetic system. Indeed, excellent work on oscillatory swimmers has been conducted, but many of the autonomous oscillatory swimmers have been metallic particles utilizing hydrogen peroxide fuel.^25−27,34−36^ In the cases of the study of oscillatory soft structures, the focus has been on individual structures.^40−45^

In this work, we study the interactions of autonomous soft swimmers, powered by the nonlinear oscillatory Belousov–Zhabotinsky (BZ) reaction, which utilizes an organic fuel and was originally developed to mimic the metabolic Kreb’s cycle.^46,47^ These swimmers undergo a nonfully reciprocal oscillatory motion yielding a net displacement due to the asymmetric nature of the BZ waves on the gel. The swimmers are made from BZ responsive hydrogels that covalently incorporate the catalyst of the BZ reaction within the hydrogel backbone.^48−50^ When placed in a solution containing BZ reactants the swimmers undergo motion.^43−45^ In this work we interrogate the interactions and resultant dynamics of multiple BZ swimmers. This work is a starting point for the design of collective behaviors utilizing autonomous chemically propelled soft swimmers.

## 2 Experimental Section

### 2.1 BZ Responsive Hydrogel Synthesis

BZ responsive hydrogels are composed of a poly *N*-isopropylacrylamide (PNIPAM) backbone cross-linked with *N*,*N*-methylene bis(acrylamide) cross-linker and where the BZ catalyst ruthenium tris(2,2-bipyridine), Ru(bpy)_3_, is covalently incorporated into the gel via a vinyl group. To enhance the elastic properties of the gel, 2-acrylamido-2-methylpropanesulfonic acid (AMPS) is incorporated into the formulation.^43−45^ A 1 mm thick gel sheet was synthesized. Details of the synthesis procedure of the poly(NIPAM-*co*-Ru(bpy)_3_-*co*-AMPS) gel can be found in the Supporting Information. The gel sheet was then cut to produce circular disc swimmers 8 mm and 4 mm in diameter. Millimeter sized structures were selected to allow the visualization of the red-green oscillatory BZ chemical waves on the gels, as well as allow wave confinement, as waves are on the same order of length. The catalyst in the gel is red in the reduced Ru(II) state and green in the oxidized Ru (III) state. The gel oscillates between these two states and their respective colors with the progression of the oscillatory BZ reaction. These states are seen as red-green waves propagating in the gel.

### 2.2 Experimental Setup, Video Capture, and Tracking

Swimmers are put in a catalyst-free BZ solution containing: 0.0625 M malonic acid, 0.084 M NaBrO3 and 0.894 M nitric acid.^43−45^ The PNIPAM-based hydrogel material used for the synthesis of swimmers is less dense than water and the catalyst-free BZ solution, which has a density of 1.042 gmL^–1^. As such the gel sits at the air–liquid interface, 5 mm above the bottom of the container and hence 5 times the height of the gel swimmer.^43−45^ Swimmer motion is observed in a 5 cm glass Petri dish at a constant temperature of 19 ± 1 °C. Motion is captured in top view using a Canon Vixia HF R80 camera at 30 frames per second. Tracking of motion is carried out using ImageJ within a 1.25 cm radius of the center of the dish to exclude any potential effects of edges. ImageJ is used to determine the *X*,*Y* positions of the swimmer in each frame, as well as determine the swimmer green level, which is extracted from the RGB images and is indicative of the oxidation level of the swimmer. Motion is captured for at least 9 h. Observations of motion reported are on at least three to six replicate samples and experiments for each experimental setup.

## 3 Results and Discussion

BZ gel swimmers undergo nonfully reciprocal motion in conjunction with the oxidation–reduction cycles of the BZ reaction resulting in a net displacement.^43−45^ This motion correlates with the color changes observed in the gel.

### 3.1 Individual Swimmers

In our previous work, we demonstrated that BZ gels unhindered by the presence of container edges, undergo nonfully reciprocal, oscillatory net displacement. The direction and speed of this displacement can be controlled by the shape of the gel swimmer.^43^ In the case of circular gels, the direction of motion is determined by the location of BZ wave initiation. BZ swimmers operate in the low Reynolds number regime as determined by eq 1
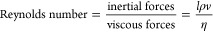
1

For a circular 4 mm diameter gel swimmer, *l* is the characteristic length of the swimmer, taken to be its diameter, *v* is the swimmers’ average velocity at 1.0 × 10^–5^ ms^–1^, ρ is the density of the fluid measured to be 1.042 g mL^–1^, and η is the viscosity of the aqueous solution as approximated to that of water at 8.9 × 10^–4^ Pa·s. The Reynolds number is calculated to be 5 × 10^–2^. This is a low Reynolds number system where viscous forces dominate over inertial forces.

Yet, despite operating in the low Reynolds number regime, all gels, including fully symmetric circular gels, can undergo a net displacement. This is due to the asymmetric nature of the BZ wave propagating within the gel and driving motion; as well as the asymmetric difference between the rate of the oxidation reaction causing forward propulsion and the reduction reaction leading to propulsion in the reverse direction. These two aspects of asymmetry within the low Reynolds number system result in the overcoming of the scallop theorem and result in a net displacement. The direction of this displacement tends to be toward the initiation point of the oxidation wave.

The propulsion force in this low Reynolds number regime is equivalent to the drag force experienced by the swimmer and is evaluated using eq 2 below.^43,51,52^
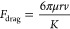
2μ is the dynamic viscosity of liquid taken to be that of water at 1 × 10^–3^ kg(ms)^−1^, *r* is the radius of the equivalent volume sphere for the swimmer at 1.44 × 10^–3^ m, *v* is the swimmers’ average velocity at 1.0 × 10^–5^ ms^–1^ and *K* is the shape factor that takes into consideration the shape of the structure, for the orientation of motion and the dimensions of the swimmer this approaches 1.^43^ The drag force is 270 pN.

### 3.2 Swimmer Interactions

**Figure 1:**
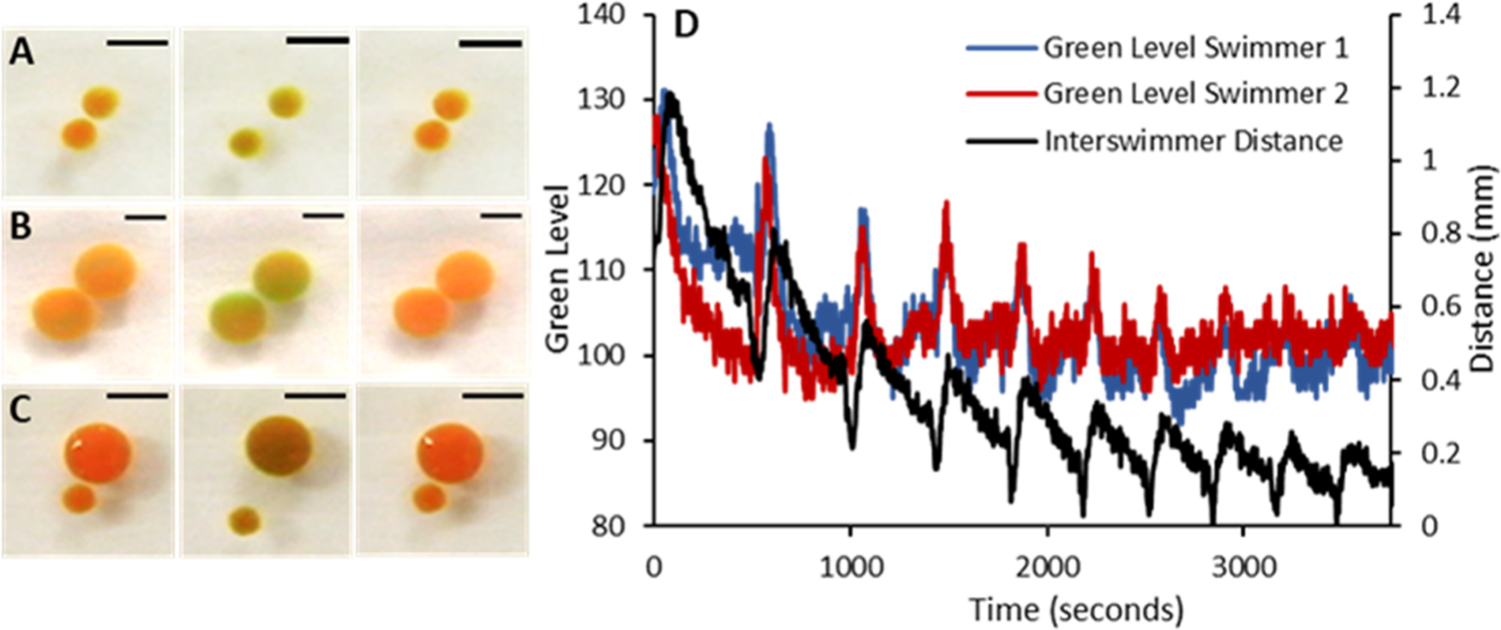
Inter swimmer Distance and Oxidation State: Frame sequences of swimmers going through a cycle from reduced to oxidized back to the reduced state and their associated motion. An increase in inter swimmer separation is observed for the oxidized state as seen in the second frame of the series in (A–C). (A) Two 4 mm diameter disc swimmers. Scale bar 10 mm. Times: 0, 32, 68 s. (B). Two 8 mm diameter disc swimmers. Scale bar 5 mm. Times: 0, 217, 400 s. (C). One 4 mm and one 8 mm diameter disc swimmers. Scale bar 10 mm. Times: 0, 120, 300 s. (D). Representative plot of the oxidation level of swimmers, indicated by the Green Level (blue and red lines, left *y*-axis), and the Inter swimmer Distance (black line, right *y*-axis) as a function of time for two 8 mm diameter swimmers. A clear correspondence between peaks in the Green Level and Inter swimmer Distance exists.

To understand the interactions between swimmers, two swimmers at a time were first interrogated. Swimmers were found to undergo a cyclic increase and decrease in their inter swimmer distance correlated with the oxidation state of the swimmers. When swimmers are in the green Ru(III) oxidized state, there is a corresponding increase in interswimmer distance, while in the red Ru(II) reduced state interswimmer distance decreases. This is observed for swimmers of different sizes as well as swimmers of mixed sizes. Figure 1 (Videos S1, S2, and S3).

The oscillation in the magnitude of inter swimmer distance is indicative of a competition between inter swimmer attractive and repulsive forces, where the relative level of attraction or repulsion is dependent upon the oxidation state of the swimmer. The oscillatory oxidation and reduction of the swimmers results in an oscillatory change in the inter swimmer distance correlated with the BZ cycle.

Attraction between swimmers originates from lateral capillary forces. Contributing to the attractive lateral capillary forces are primarily lateral immersion forces due to swimmer wettability.^53−55^ The capillary immersion force between two gel swimmers at the air–liquid interface is dependent upon the contact angle of the fluid with the gel, namely its wettability, which is dependent on its oxidation state. The magnitude of this force can be determined using eq 3.^56−58^
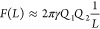
3where *Q* is evaluated using eq 4
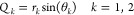
4where θ_1_ and θ_2_ are the contact angles of the liquid with the first and second gel swimmer, respectively, γ is the surface tension of the liquid, *L* is the distance between the swimmers, where this equation is applied to interparticle distances smaller than the capillary length which is given by eq 5.^56^
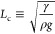
5where ρ is the density of the liquid and *g* is the acceleration due to gravity.^56^

The contact angle for the reduced and oxidized states are as reported in the literature as 48 ± 3° for the reduced state and 42 ± 3° for the oxidized state.^45^ Evaluating eq 4, where 40 mN m^–1^ is the surface tension of the catalyst-free BZ solution with a capillary length over 2 mm, and L is taken to be the maximal separation distance of two 8 mm diameter swimmers at 1.19 mm (Table 1), yields a force of 1.9 mN for two swimmers in the reduced state and 1.5 mN for two swimmers in the oxidized state. In both cases the attractive interaction is large enough to overwhelm the propulsive force, ensuring that the separate swimmers remain within each other’s influence. The capillary interaction is attractive regardless of the oxidation state of the swimmers, albeit smaller in the case of swimmers in the oxidized state. Hence when both swimmers are in the reduced state the decrease in the inter swimmer distance that occurs can be attributed to lateral capillary interactions. Yet the increase in inter swimmer distance when one or both swimmers is in the oxidized state cannot be explained by capillary interactions which continue to be attractive.

**Table 1:** Maximum Separation in Millimeters and % Body Length between Swimmers of Different Sizes

disc 1 diameter (mm)	disc 2 diameter (mm)	maximum separation (mm)	% body length
8	8	1.19 ± 0.1	15
4	4	2.27 ± 0.1	57
8	4	2.75 ± 0.3 (out of phase)	46
		4.07 ± 0.2 (in phase)	68

Upon the oxidation of both or one of the gel swimmers from the Ru(II) state to the Ru(III) state, the ruthenium catalyst incorporated into the gel goes from a 2+ charge to a higher 3+ charge, and there is a significant increase in the positive charge on the swimmers.^48−50^ Along with this increase in positive charge, an increase in inter swimmer separation is observed. This increase in distance originates from electrostatic repulsive forces between the more highly positively charged swimmers, as is typically found for like-charged particles.^53,54^ ζ-Potential measurements of the gel under BZ reaction conditions reported in the literature indicate that in the oxidized state the gel has a net positive charge with a reported ζ-potential of +3.5 mV.^59^ The density of the gel swimmers is lower than that of the BZ solution, resulting in a gel that is located at the air–liquid interface. For the portion of the swimmer immersed within the liquid, charges are completely screened by the ions in the catalyst-free BZ solution that has a pH < 1. Hence the Debye screening length is below a nanometer and significantly smaller than the inter swimmer distance. For the portion of the of the swimmers that are in air, like charges experience an electrostatic repulsion, similar to a dipole–dipole interaction. The essence of electrostatic repulsion between two like charged particles can be simplified to eq 6(54)
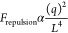
6where *q* is the total surface charge and *L* is the distance between two particles. Hence in the oxidized state the *q* value increases, increasing the repulsive force, that is observed as an increase in inter swimmer separation.

**Figure 2:**
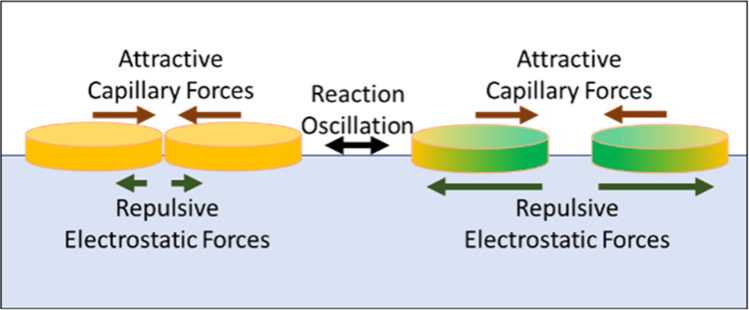
Schematic of the forces at work between BZ swimmers. Forces are represented by arrows indicating the direction and magnitude of the force. The red arrows represent the attractive lateral capillary forces. The green arrows represent electrostatic repulsive forces. The leftmost schematic shows both swimmers in the reduced state. The rightmost schematic shows the swimmer is in the green oxidized state.

Hence attraction between swimmers originates from lateral capillary forces and the repulsion originates from electrostatic repulsive forces. Figure 2. The relative size of these competing forces is dependent upon the oxidation state of the swimmer. When one or more of the swimmers in the higher charge oxidation state, the increase in charge results in the dominance of repulsive forces and an increase in the inter swimmer distance. Upon returning to the lower charge reduced state Ru(II) there is a decrease in the electrostatic repulsion, and attractive capillary forces dominate resulting in a decrease in the inter swimmer distance.

Swimmers that are smaller in size exhibit a larger interparticle separation. This larger separation is due to increased repulsion due to their higher surface charge given the higher surface-to-volume ratio of smaller swimmers. Indeed, maximum inter swimmer distances for the smaller 4 mm diameter swimmers can reach up to a magnitude of 57% of the swimmer body length as compared to 15% for 8 mm diameter disks. Table 1. Additionally, the attractive lateral immersion capillary force between two swimmers is directly proportional to the size of the particle.^53^
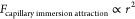
7where *r* is the radius of the particle or its equivalent volume sphere. The simultaneous increase in the repulsive force and reduction in attractive capillary force contribute to the increased maximum inter swimmer separation for the smaller 4 mm diameter swimmers.

Moreover, the higher magnitude drag forces on the larger swimmers also result in a smaller interparticle separation during repulsion.

**Figure 3:**
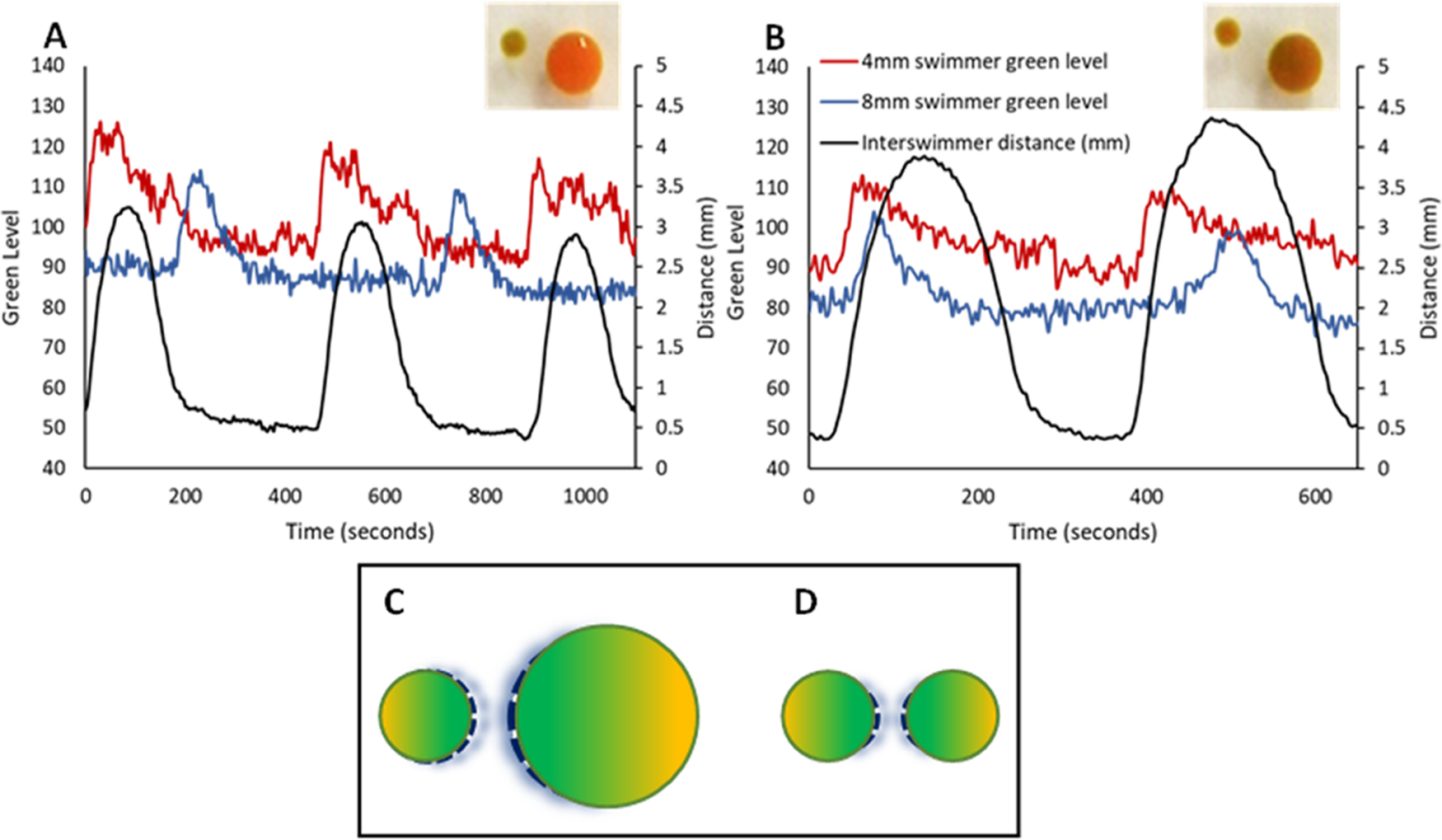
(A). Plot of the oxidation green levels of out of phase 4 mm diameter and 8 mm diameter swimmers, (blue and red lines, left *y*-axis), and the Inter swimmer Distance (black line, right *y*-axis) as a function of time. Inset: Representative image of out of phase 4 mm diameter and 8 mm diameter swimmers. (B). Plot of the oxidation green levels of in phase 4 mm diameter and 8 mm diameter swimmers, (blue and red lines, left *y*-axis), and the Inter swimmer Distance (black line, right *y*-axis) as a function of time. Inset: Representative image of in phase 4 mm diameter and 8 mm diameter swimmers (C). Surface of exposure between a 4 mm and 8 mm diameter swimmer. Black dotted line. (D). Surface of exposure between two 4 mm diameter swimmers which tends to be smaller. Black dotted line with blue shading.

Interestingly, when a larger 8 mm diameter swimmer and a smaller 4 mm diameter swimmer are mixed the maximum inter swimmer distance occurs when the swimmers’ oxidation–reduction cycles are in phase. This can be attributed to the larger repulsive surface encountered by the smaller swimmer. So, although a smaller swimmer has a larger relative surface charge, a larger counterpart means that the smaller swimmer is in contact with a larger absolute surface with a positive charge and hence can move further away during the repulsive interaction. This is schematically represented in Figure 3. Maximum repulsion and hence inter swimmer distance is observed for swimmers that are in phase because both have higher net charge resulting in stronger electrostatic repulsion. Yet even out of phase swimmers exhibit repulsion with the effect being more magnified when the smaller swimmer is in be oxidized state due to its higher relative surface charge. Figure 3.

### 3.3 Interparticle Separation: Three and More Swimmers

**Figure 4:**
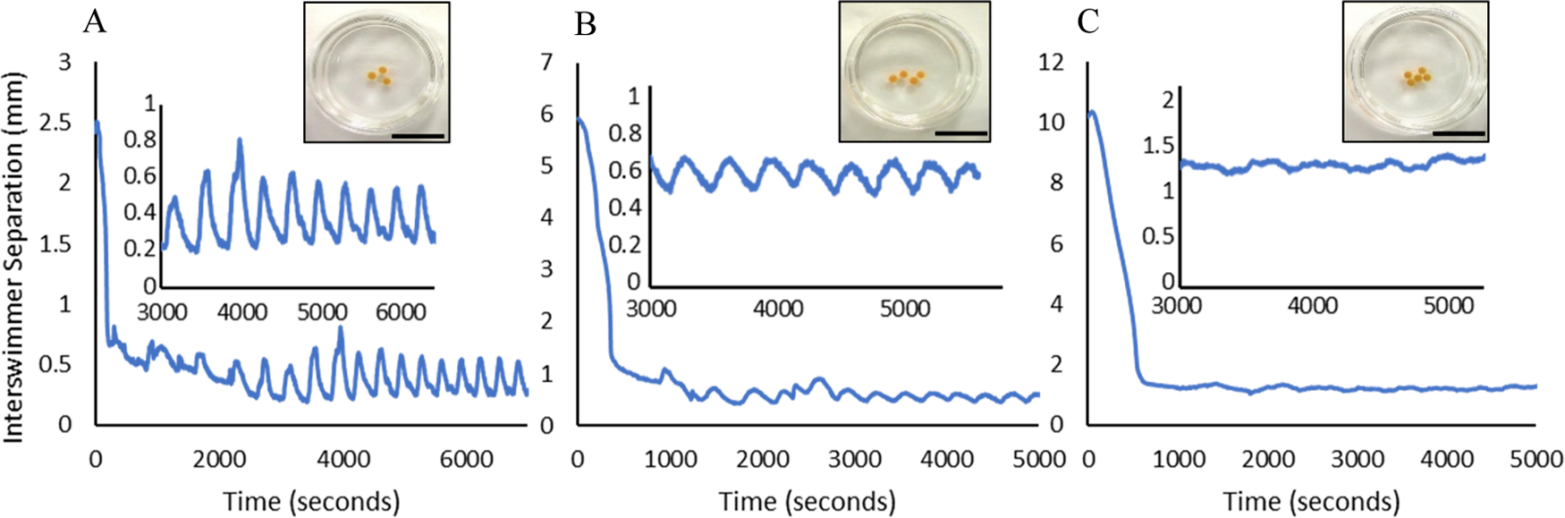
Inter swimmer separation as a function of time for multiple swimmers. (A) Three 4 mm diameter swimmers. Inset: zoomed in portion on region of the plot and image of experimental setup. Scale bar: 25 mm. (B) Four 4 mm diameter swimmers. Inset: zoomed in portion on region of the plot and image of experimental setup. Scale bar: 25 mm. (C) Five 4 mm diameter swimmers. Inset: zoomed in portion on region of the plot and image of experimental setup. Scale bar: 25 mm.

Oscillatory increase and decrease in inter swimmer separations is also observed when three and more swimmers are placed together. To quantify the average, inter swimmer distance, the center of mass for each frame is calculated, the distance from each swimmer to the center of mass is determined, and the average of these distances is taken, using eq 8.^36^
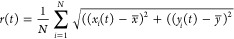
8When the distance between each sample and the center of mass is determined and those distances averaged and plotted as a function of time, an oscillation in the distance is observed, indicating a pulsation of the swimmers around the center of mass Figure 4.

As the number of swimmers increases, there is a reduction in the amplitude of the pulsation oscillation. This is because the pulsation is not necessarily symmetrical about the center as the number of nearest neighbors for each swimmer increases. Hence attractive and repulsive forces applied to a swimmer due to adjacent swimmers may occur in opposing directions reducing its overall amplitude of motion. Hence, while some swimmers may be in the green oxidized state, others may be in the red reduced state, yielding attraction and repulsion simultaneously for different entities within the group, and in different locations. Hence attraction among some swimmers cancels out repulsion among others, and we see a reduction in the amplitude of the oscillation. This results in an overall smaller amplitude.

**Figure 5:**
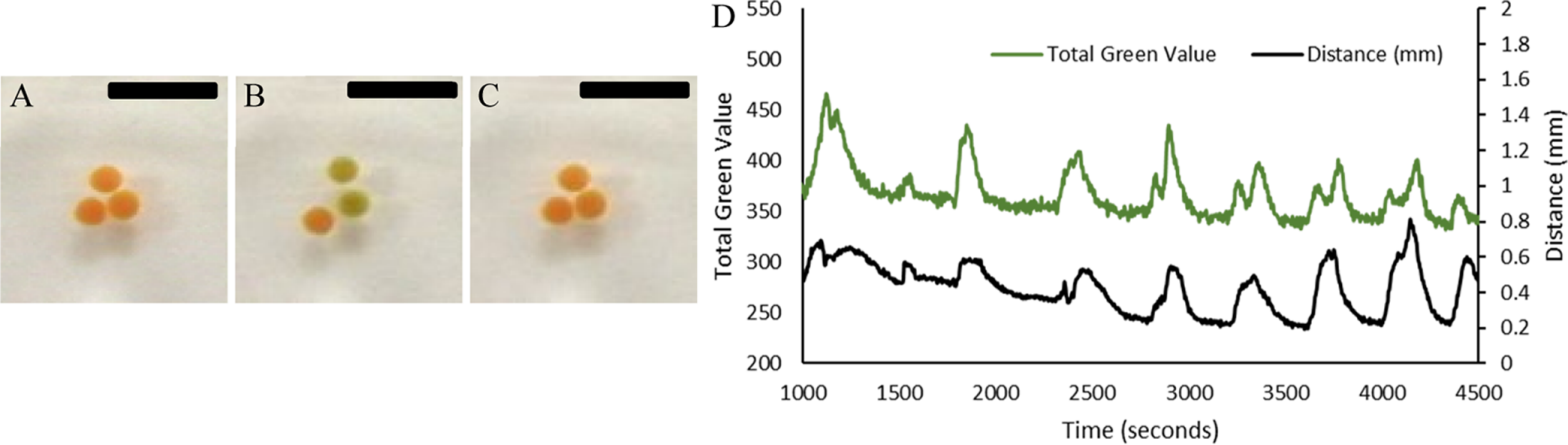
(A–C) Frame sequence demonstrating pulsation of multiple swimmers as they go from the reduced to the oxidized, back to the reduced state. Times: 0, 187, 367 s. Scale bar: 15 mm. (D) Plot of the total green level as a function of time for three swimmers. (green line, left *y*-axis); and the Inter swimmer Distance (black line, right *y*-axis) as a function of time. There is a clear correlation between the overall oxidized state of the swimmers and the inter swimmer distance.

In general, peaks in swimmer separation for multiple swimmers correlates with the total green value of the multiple swimmers. This can be seen in Figure 5.

**Figure 6:**
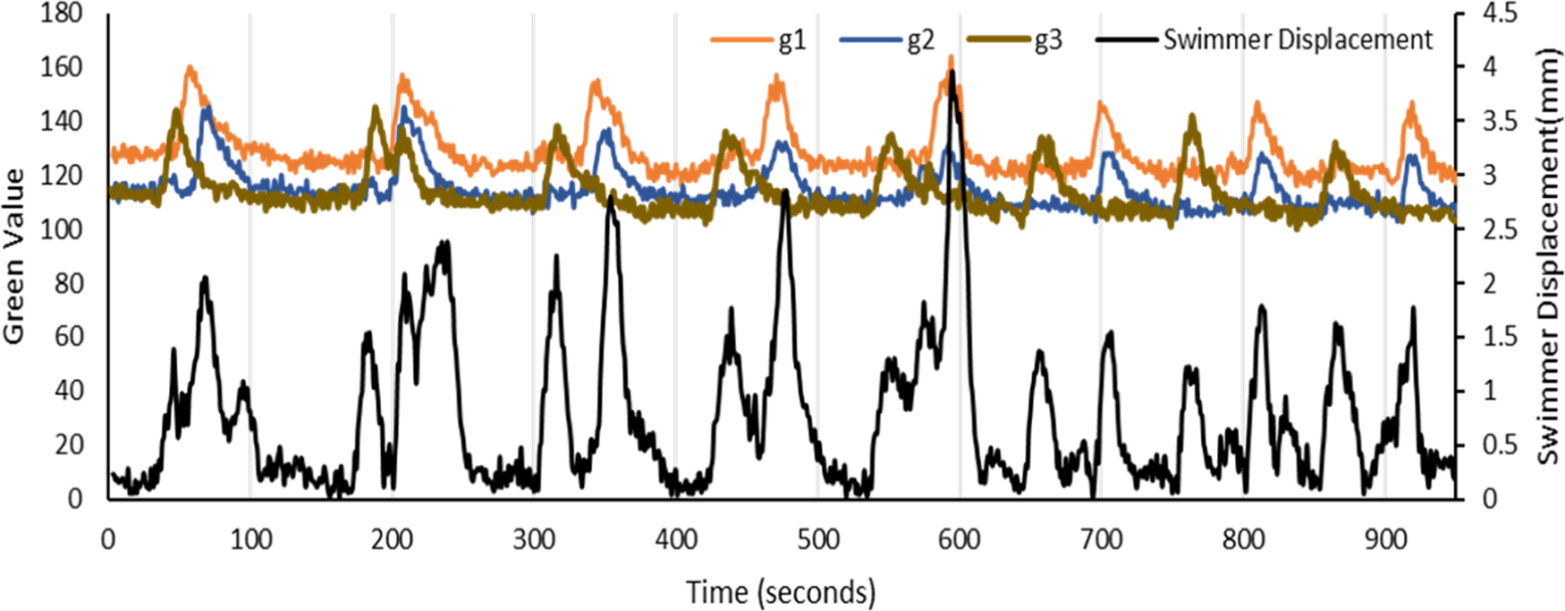
Plot of the oxidation level of swimmers, indicated by the Green Level (orange line for swimmer 1, blue line for swimmer 2 and brown line for swimmer 3, left *y*-axis), and the displacement of swimmer 1 (black line, right *y*-axis) as a function of time for three 4 mm diameter swimmers. A clear correspondence between peaks in the Green Level of the swimmers and displacement of swimmer 1 is apparent, with the maximum displacements corresponding to the maximum green level of swimmer 1.

When the color and displacement of individual swimmers within a group of multiple swimmers is tracked, swimmer displacement increases with the increase in the oxidation level of either the swimmer itself, or that of the oxidation level of any of the adjacent swimmers; with the maximum displacement occurring when the swimmer itself is in the oxidized state. This can be seen in Figure 6, where a swimmer’s displacement is plotted along with the oxidation levels of each of the three swimmers, as indicated by their green level. When the green level of swimmer one (orange line) is at a maximum, the displacement of swimmer one (black line) is also at a maximum. Interestingly, even when swimmer one is in the reduced oxidation state, indicated by a low green level, it experiences an increase in displacement if any of the adjacent swimmers is in their peak oxidized (green) state. When swimmer one is in phase synchrony with any of its neighbors, it experiences a larger displacement than when it is not in phase. This is again due to the larger positive charge on both swimmers and the resulting increase in repulsion.

### 3.4 Chemical Communication between Swimmers

BZ entities are capable of chemical communication as demonstrated by work on static BZ droplet in a fixed organization.^60−63^ This communication is mediated by chemicals released during the progress of the BZ reaction. These chemicals include excitatory chemicals, such as bromine dioxide and bromous acid, as well as inhibitory chemicals such as bromine.^60−63^ Excitatory chemicals typically yield in phase synchronization, while inhibitory chemicals result in antiphase synchronization where entities are 180 deg out of phase. In this closed system both chemicals are released and there is no attempt to selectively eliminate either.

**Figure 7:**
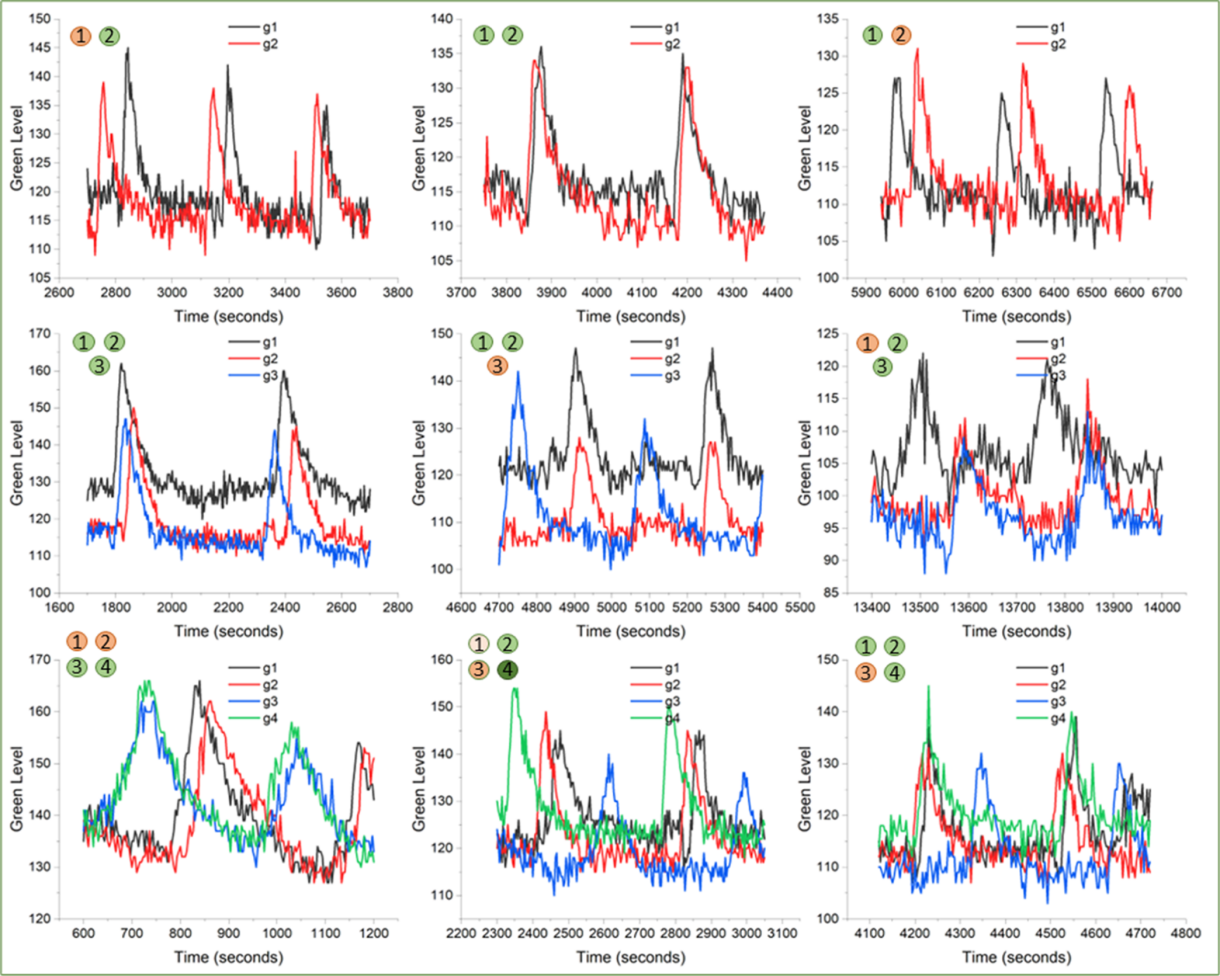
Plots of oxidation level, as indicated by green level, as a function of time. Green levels of individual swimmers are represented by different colors as indicated in the legend. Top panels represent oxidation levels for two swimmers. Left to right: two swimmers not in phase; two swimmers in phase; two swimmers not in phase with the reversal of firing order where firing refers to the appearance of the green oxidation wave. Middle panels represent oxidation levels for three swimmers, Left to right: all swimmers in phase; one combination of two swimmers in phase (1 and 2); another combination of two swimmers in phase (2 and 3). Bottom panels represent oxidation levels for four swimmers, Left to right: two sets of swimmers in phase; no swimmers in phase; three swimmers in phase.

In this system, swimmers of similar size typically start oscillating in phase when they contact the catalyst free BZ solution simultaneously. Overtime minor differences in frequency between swimmers, potentially due to microscale variations in size or statistically random variations in chemical concentrations surrounding the swimmers, results in phase drifting between swimmers. This results in swimmers gradually, over several cycles, going into and out of phase with time. Interestingly, as the number of swimmers increases beyond two, all potential permutations of in phase and out of phase relationships between different swimmers is observed. For example, for three swimmers, 1, 2, and 3 the synchrony relationships observed include: all in phase, two swimmers in phase at a time including: 1 and 2 in phase with number 3 out of phase; 2 and 3 in phase with number 1 out of phase; 1 and 3 in phase with number 2 out of phase, as well as none of the swimmers in phase. For four swimmers, additional permutation include different combinations of three of the swimmers oscillating in phase with a single swimmer not in phase, as well as, 2 sets of 2 swimmers oscillating in phase with each other but not the other 2. These various states of synchrony can be seen in Figure 7.

## 4 Conclusions

In this work we report the dynamic behavior and interactions between chemically actuated soft swimmers that rely on the breakdown of an organic fuel in the form of the nonlinear BZ reaction. It is found that BZ swimmers, upon approaching each other, undergo oscillatory attraction and repulsion in conjunction with the BZ reaction. Repulsion is associated with the highly charged oxidized state of the swimmer and is attributed to electrostatic repulsion between positively charged BZ swimmers. While attraction occurs when swimmers are in the lower charged reduced state and is attributed to lateral capillary attractive forces between swimmers. Smaller swimmers exhibit larger separations during repulsion due to higher surface charge given their larger surface-to-volume ratio, lower lateral capillary attractive forces as well as lower drag forces upon motion. In phase swimmers also exhibit larger inter swimmer distances upon repulsion when both are in the oxidized state due to the higher charge on each swimmer. When more than two swimmers interact an oscillation in their overall separation occurs. For these pulsating groups of swimmers, there is a correlation between the total green level of the swimmers and the inter swimmer distances with larger distances occurring at higher total green levels, where the green level is indicative of the more highly charged oxidized state. The amplitude of this pulsation decreases as the number of swimmers increases. This is because the phase relationships between multiple swimmers varies more broadly resulting in some swimmers being in the reduced state and undergoing attraction, while others being in the oxidized state and undergoing repulsion simultaneously. This cancels out the net effect on the amplitude of oscillation. Individual swimmers within a group exhibit larger displacements correlated with their oxidized state as well as with the oxidized states of their adjacent neighbors even when they are in the reduced state. Multiple swimmers exhibit several states of chemical synchrony that represents all potential permutations of in phase and out of phase combinations.

Overall, this work provides a model system for the interaction of biomimetic soft swimmers. The swimmers are autonomous and propel by the consumption of an organic fuel utilizing a nonlinear chemical reaction. Knowledge of the interactions of multiple soft swimmers serves as the starting point for the design and engineering of controlled collective behaviors for the accomplishment of various functions.

## Supporting Information Available

Additional experimental details and materials for gel synthesis (PDF)
Gel robot motion, 2 swimmers, 4 mm diameter each (AVI)
Gel robot motion, 2 swimmers, 8 mm diameter each (AVI)
Gel robot motion, 2 swimmers, a 4 mm diameter swimmer and a 8 mm diameter swimmer (AVI)
Gel robot motion, 3 swimmers, 4 mm diameter each (AVI)
Gel robot motion, 4 swimmers, 4 mm diameter each (AVI)
Gel robot motion, 5 swimmers, 4 mm diameter each (AVI)

## Supplementary Material

jp4c07069_si_001.pdf

jp4c07069_si_002.avi

jp4c07069_si_003.avi

jp4c07069_si_004.avi

jp4c07069_si_005.avi

jp4c07069_si_006.avi

jp4c07069_si_007.avi
