# Supplementary material for: Interactions and Oscillatory Dynamics of Chemically Powered Soft Swimmers
Source: J Phys Chem B. 2024 Dec 23;129(1):554–62. doi: 10.1021/acs.jpcb.4c07069 (PMC11726663; doi:10.1021/acs.jpcb.4c07069)
Supplement: Supplementary file 1 — jp4c07069_si_001.pdf [file jp4c07069_si_001.pdf]

Supporting Information

Interactions and Oscillatory Dynamics of Chemically Powered Soft Swimmers

Suzanne Ahmed[1] and Juan Perez-Mercader*[2,3]*

[1]Department of Nanoscience, Joint School of Nanoscience and Nanoengineering, University of North Carolina at Greensboro, 2907 E Gate City Blvd, Greensboro, NC 27401

[2]Department of Earth and Planetary Sciences and Origins of Life Initiative, Harvard University, 20 Oxford Street, Cambridge, MA 02138, USA.

[3]Santa Fe Institute, Santa Fe, NM 87501, USA.

* Corresponding Author, Email: saahmed2@uncg.edu, jperezmercader@fas.harvard.edu

Table of Contents

Videos of Gel Swimmer Motion……………………………………………………………...S-1

Detailed Experimental Section………………………………………………………………….S-2

Supporting Videos: Gel Robot Motion

Video S1: 2 swimmers, 4 mm diameter each

Motion of 2 swimmers, each 4 mm in diameter in a catalyst-free BZ solution. Motion occurs in a 5 cm diameter glass Petri dish. Motion that occurs at the center of the Petri dish away from the edge is presented. (video rate: 100 times as fast as the actual speed).

Video S2: 2 swimmers, 8 mm diameter each

Motion of 2 swimmers, each 8 mm in diameter in a catalyst-free BZ solution. Motion occurs in a 5 cm diameter glass Petri dish. Motion that occurs at the center of the Petri dish away from the edge is presented. (video rate: 100 times as fast as the actual speed).

Video S3: 2 swimmers, a 4 mm diameter swimmer and a 8 mm diameter swimmer

Motion of 2 swimmers, each 8 mm in diameter in a catalyst-free BZ solution. Motion occurs in a 5 cm diameter glass Petri dish. Motion that occurs at the center of the Petri dish away from the edge is presented. (video rate: 100 times as fast as the actual speed).

Video S4: 3 swimmers, 4 mm diameter each

Motion of 3 swimmers, each 4 mm in diameter in a catalyst-free BZ solution. Motion occurs in a 5 cm diameter glass Petri dish. Motion that occurs at the center of the Petri dish away from the edge is presented. (video rate: 100 times as fast as the actual speed).

Video S5: 4 swimmers, 4 mm diameter each

Motion of 4 swimmers, each 4 mm in diameter in a catalyst-free BZ solution. Motion occurs in a 5 cm diameter glass Petri dish. Motion that occurs at the center of the Petri dish away from the edge is presented. (video rate: 100 times as fast as the actual speed).

Video S6: 5 swimmers, 4 mm diameter each

Motion of 5 swimmers, each 4 mm in diameter in a catalyst-free BZ solution. Motion occurs in a 5 cm diameter glass Petri dish. Motion that occurs at the center of the Petri dish away from the edge is presented. (video rate: 100 times as fast as the actual speed).

Detailed Experimental Section

Swimmer synthesis. BZ responsive, poly (NIPAM−*co*-$Ru(bpy)_3$−*co*-AMPS) hydrogel was synthesized using a procedure based on established methods. [1-3]

N-isopropyl acrylamide monomer (NIPAM, 6.24 x10^{-1}g), methylene bisacrylamide (1.12x10^{-2}g), Ruthenium tris(2,2′- bipyridine) with a vinyl group, namely: Ruthenium(II)(4-vinyl-4′-methyl-2,2′- bipyridine)bis(2,2′bipyridine) bis(hexafluorophosphate) (4.6x10^{-2}g) and azobisisobutyronitrile initiator (AIBN, 2.7x10^{-2}g) were mixed in 2mL of methanol. 2- acrylamido-2-methylpropanesulfonic acid (AMPS, 2.2 x10^{-2}g) was dissolved in 1.6 mL of distilled water and 0.4 mL of dimethyl sulfoxide. The two solutions were then mixed. All chemicals were obtained from Sigma-Aldrich except the Ruthenium(II)(4-vinyl-4′-methyl-2,2′-bipyridine)bis(2,2′bipyridine) bis(hexafluorophosphate) which was obtained from Synthon Chemicals GmbH & Co. KG. The mixture was bubbled with nitrogen for 20 minutes. After that the solution was injected between two glass slides with a PDMS spacer 1mm in thickness. It was then put in an oven at 65°C for 18 hours. The hydrogel was then rinsed for 14 days , first in 100% ethanol then in gradually decreasing amounts of ethanol and increasing amounts of distilled water until, prior to carrying out any experiments, the gel was rinsed in pure distilled water for two days. Rinsing was done to get rid of any unreacted monomer and catalyst species. Circular gel cuts 8mm and 4mm in diameter were then made to generate the gel swimmers.

References:

(1) Ahmed, S.; Perez-Mercader, J. Autonomous Low-Reynolds-Number Soft Robots with Structurally Encoded Motion and Their Thermodynamic Efficiency. *Langmuir* **2021**, *37* (27), 8148–8156. https://doi.org/10.1021/acs.langmuir.1c00765.

(2) Yoshii, M.; Yamamoto, H.; Sumino, Y.; Nakata, S. Self-Oscillating Gel Accelerated While Sensing the Shape of an Aqueous Surface. *Langmuir* **2016**, *32* (16), 3901–3906. https://doi.org/10.1021/acs.langmuir.6b00337.

(3) Nakata, S.; Yoshii, M.; Suzuki, S.; Yoshida, R. Periodic Reciprocating Motion of a Polymer Gel on an Aqueous Phase Synchronized with the Belousov–Zhabotinsky Reaction. *Langmuir* **2014**, *30* (2), 517–521. https://doi.org/10.1021/la403675z.
